# Religion and nationalism revisited: Insights from southeastern and central eastern Europe

**DOI:** 10.1177/14687968231207980

**Published:** 2023-10-23

**Authors:** Anna Triandafyllidou

**Affiliations:** Canada Excellence Research Chair in Migration and Integration, 177416 Toronto Metropolitan University, Toronto, ON, Canada

**Keywords:** Identitarian, politicising religion, populism, post-communist, religious diversity, religious nationalism, state-church relations

## Abstract

This paper explores the dynamics behind the rise of religious nationalism in Central Eastern and Southeastern Europe with distinct populist, nativist, and authoritarian overtones. The paper explores the relationship between nationalism and religion today and the broader transformation challenges both within the region and more globally that can shape this relationship. It then looks closer into the historical experiences in the region with regard to the relationship between state and church as well as nationalism and religion, critically analysing how these relations have evolved during nation-state formation in the 19th and early 20th century, under Communism, and in the last three decades. Analysing critically the relevant literature, the paper discusses the entanglements between state and religious institutions as well as between national identity and faith, and how these are mobilised today. The paper argues for the need to consider both internal and external factors in the evolution of the relationship between nationalism and religion in Central Eastern and Southeastern Europe and more broadly.

## Introduction

Southeastern and Eastern Europe have historically been recognised as ‘home’ to ethnic forms of nationalism, based on beliefs in common descent, common ethnicity and culture, and relatively low tolerance to cultural and religious diversity. Indeed the term ethnic unmixing (Brubaker, 1998) has been coined to refer to the processes of creating ethno-cultural homogeneity within national states in the region. At the same time the region, particularly Southeastern Europe, has been characterised by relatively strong attachment to religion, which has often been closely intertwined with national identity acting as an important marker between minorities and majority within each country. With the exception of Greece, all countries have been part of what was once the Warsaw Pact and have experienced a communist and highly secular regime for decades. After 1989, this was followed by a revival of both religion and national identity, as well as of the relationship between the two, albeit tamed to adapt to minority rights and integration within the Council of Europe and the European Union.

During the late 2010s and early 2020s we are witnessing yet another turn in these countries with an increased anti-immigrant sentiment. Islamophobia and nationalist rhetoric have risen even if opinion polls suggest that people in the region are not particularly attached to religion. This paper takes stock of this historical and contemporary context to reflect on the nature of the relationship between nationalism and religion in Southeastern and Central Eastern Europe today. The paper starts with an analytical reflection on the nature of nationalism as a late modern phenomenon and discusses its dynamics, in general and specifically in relation to religion. The paper argues that while both nationalism and religion seek to offer responses to socio-political and existential inquiries about the past, present, and future of both individual and community, they can be seen as antithetical since nationalism celebrates the universalism of the particular (the nation) while religion offers a broader scope of universalism (the faith is open to everyone). I argue that in late modernity, religion becomes particularly important to nationalism as a boundary, helping to (re)define the nation and differentiate from internal and external others as well as to anchor the nation in old solidities. Having said this, religion takes also a specific form and meaning in Central Eastern and Southeastern Europe post-1989 as it becomes emblematic of the political transition and gets mobilised by different political forces as a means of both uniting but also differentiating among nations and countries.

Taking stock of the relevant literature, this paper explores the dynamics behind the rise of religious nationalism in Central Eastern and Southeastern Europe with distinct populist, nativist, and authoritarian overtones. It examines the relationship between nationalism and religion today and the broader transformation challenges within the region and more globally that can shape this relationship. The paper then looks closer into the historical experiences in the region with regard to the relationship between state and church as well as nationalism and religion, critically analysing how these relations have evolved during nation-state formation in the 19th and early 20th century, under Communism and in the last three decades.

Critically reviewing the relevant literature, the paper discusses the entanglements between state and religious institutions as well as between national identity and faith. It investigates how these are mobilised today by states to respond to the challenges of a prolonged and painful socio-economic transformation in the absence of ideological confrontation that characterises the post-1989 period and in the context of a strong institutional entanglement, particularly between Orthodox Churches and state power. The paper argues for the need to consider both internal and external factors in the evolution of the relationship between nationalism and religion in Central Eastern and Southeastern Europe and more broadly. This study focuses on the institutional and political aspects of state religious institutions’ relationships and does not cover aspects of individual or community mobilisation, beliefs, or feelings about the same that have been covered in other studies quite aptly (Gauthier 2022; Pickel and Sammet 2012).

## Nationalism and religion in the 21st century

It has been argued that the classification of nations between those predominantly ethnic and those predominantly civic has run its course (Triandafyllidou, 2020). The reason is not only that both ethnic and civic arguments can be used to exclude minorities or migrants and create distance or enmity between different nations (Triandafyllidou, 2001), but most importantly that this distinction was predicated on different paths of state and nation formation that are no longer so important in shaping the course of new nationalisms.

Nationalism studies during the 20th century have been largely preoccupied with the question of whether nations have existed since time immemorial or whether they are products of modernity. Different historical, socio-political, and economic arguments have been put forward to defend either perspective. While each has their merits, it is my contention that eventually a synthesis provides a more appropriate account. In this sense, the emphasis that Anderson, (1981) put on the role of imagined communities as part of a wider process of industrialisation, improvement in transport and communication, the emergence of urban middle-classes, and the spread of literacy remains important. Likewise, the primordialists’ argument that nations emerge out of ethnic communities that survived and transformed through the centuries is valid too (Hastings, 1997). Eventually, the ethno-symbolist approach (Smith, 2009) offers a balanced view recognising the importance of the scientific and economic developments in the 17th and 18th centuries making the nation functional to this historical period, but also pointing to the fact that there can be no nation that is totally socially and culturally engineered without a pre-existing ethno-cultural fabric. The ethno-symbolist school convincingly posits that ethnic belonging and its political ramifications (which may be political in the wider rather than the narrower sense) is an important resource leveraged in modernity by the socio-economic forces of modernity in producing nations and nationalism.

The scholarly inquiry into the circumstances and factors driving the emergence of modern nations and nationalism in Europe in the post-18th century has shaped the classification of nations into ethnic vs civic. Ethnic nations, mainly to be found in Southeastern and Central Eastern Europe, are those whose definition is predominantly based on ethno-cultural elements such as a belief in common descent; shared historical memories; a link with historical territory that is seen as the homeland of the nation and which may not coincide with its current territory; and a common language as well as sometimes also a common religion. Civic nations, mostly located in western and northern Europe, are those whose definition privileges territorial and civic elements, notably a mass public culture, a territory and a single economy, and a set of common rights and duties among its members. Naturally language is often a pre-requisite – if anything a functional one – for civic nations too. While ethnic nations are defined as largely ascriptive (one is born into a nation), civic nations are voluntarist in their nature: a person who lives in the national territory, abides by its laws, and contributes to society and the economy for a given period of time can become a national. While these are ideal types as most nations fall somewhere in between this continuum of ethnic vs civic, these two types of nations have been historically identified with different parts of Europe.

This distinction emerged in Europe to make sense of different paths of nation-state formation. Ethnic nationalism has been largely considered to correspond to the historical experience of central, eastern and southeastern Europe, where national identities were forged in the absence of corresponding state structures and generally in opposition to empires. Civic nationalism by contrast has been identified with western European pathways to nation-state formation in countries where state structures had formed before the potent onset of modern nationalism. Within this distinction religion has been seen to play an important role, both historically and as a cultural component of a given national identity. In Southeastern and Central Eastern European nationalisms, religion emerged as an important distinctive feature as it was the type of identity that not only survived under empire but was also given a political functional role. For instance, the Ottoman Empire’s millet system privileged religion as a form of community organisation and representation. One might argue that this role of religion was relevant until the dissolution of the Ottoman and Russian empires in the early 20th century but has lost its appeal or raison d’etre in the second half of the 20th century and certainly in the 21st century.

The socio-economic developments of the last 35 years demand that we reconsider the relevance of the ethnic vs civic nationalism definitions in contemporary Europe and religion’s role in relation to nationalism. Such rethinking needs to take into account the broader societal transformations, particularly those of the last 20 years. A new geopolitical and cultural configuration in Europe (and globally) has emerged from the Communist regimes’ implosion in 1989 as well as the new power hierarchies and cultural fault line between the ‘West’ and Muslims. These societal dynamics have been further shaped by increased human migration and intensified connectivity to the point of today’s ‘virtual mobility’ through advanced digital technologies. Understanding nationalism and its relationship to religion today requires a closer attention to actual or virtual interactions with Significant Others, whether internal or external, positive or threatening (Triandafyllidou, 2020).

Whereas the 1990s had been characterised by a certain euphoria after 1989 and the reconnection of western and eastern Europe, the new century has started with profound geopolitical, cultural, and existential crises for Europe and the West. On one hand, the European Union’s ‘eastern enlargement’ and full reconnection of Europe has proved to be more difficult than originally envisaged, and hierarchies have emerged between the ‘new’ and the ‘old’ member states. On the other hand, the terrorist attacks of 9/11 and the ensuring War on Terror, violent urban protests in England (2001) and France (2005), and terrorist attacks in Madrid (2004) and London (2005) raised questions about the effective integration of post-migration minorities in European countries. Islam emerged forcefully as the new ‘civilizational’ line within Europe.

It was the very success of Eastern Enlargement – which pushed forward on the basis of political and ideological principles rather than economic considerations – alongside the emergence of international jihadist terrorism and urban tensions between post-migration minorities and native majorities that paved the way for Islam to becoming the necessary European Other. Not only had Communism collapsed and with it the Cold War geopolitical and symbolic framework, but the Central Eastern European countries were fully subscribing to the now hegemonic western European model. The Communists had been successfully ‘reformed’ – there was a need for a new Other at the European and global levels towards whom a united Europe and the Western/European values would be reaffirmed. Internally and externally, Muslims conveniently filled this vacuum; accused of creating ‘parallel societies’ within European countries, they were also framed as posing a threat to European security through terrorism. Thus, while religion had been seen as a relic of past passions, important as a cultural element but no longer relevant for political purposes, it suddenly acquired new impetus.

Although international migration has always been a feature of human history, advanced technologies and socio-political-economic dynamics within and across nations have contributed to intensified cross-border mobility of people – with different reasons and objectives (McAuliffe and Triandafyllidou, 2022). The increase in the cross-border mobility of people is a hallmark of the current era of human history. In a recent study, Recchi et al. (2019) estimate global transnational mobility at 3 billion trips annually worldwide (in the period 2011-2016) compared to an estimated 10 million migration episodes annually in the early 2010s (Abel and Sander 2014). The rise of global mobility poses both a global challenge and an opportunity: it fosters social and technological innovation but may also exacerbate social inequalities and socio-political tensions; there are also questions with regard to its environmental consequences, the potential for the spread of pandemics, and the emergence of global systemic risks (Centeno et al. 2015). Migration is acknowledged as a right (the right to emigrate, to leave one’s country), as a positive element in people’s lives (the capacity and freedom to move), and as a crisis (when people are forced to move because of a natural disaster, a war or simply the search for a better future). People move for leisure, to visit family and friends, to search for better living and working conditions, but also to seek protection. Communities may also be displaced internally or across national borders (Sassen 2014), both spatially and culturally (Dorries et al. 2022; Tomiak 2017).

In addition, migration today – and increasingly in the future – is not always or necessarily spatial or physical: it can also be virtual/digital. Advanced digital technologies do not only facilitate connections and collaborations but are also promising a new level of virtual ‘presence’ in the near future (see the recent announcement of the Metaverse by Facebook). One may borrow the term ‘saturated mobility’ from the natural sciences and consider the case of young people who may be extremely virtually mobile but physically stay put. Their mobility experience is not spatial but social and inter-subjective; through virtual mobility and connectivity, they may be experiencing the breaking down and reorganisation of social and kinship networks, as well as a level of political alienation or anti-social radicalisation as they may feel that they have lost connection with the national governments but have not found any other political actors or institutions to fill the vacuum except for online communities. On the other hand, we cannot ignore that there are many people who aspire to migrate but are not able to because they lack the resources or the right documents.

In this context, cultural and ethnic diversity has acquired a new dimension as it relates to migration, to global value chains, and to the products that we use or services we consume, including for instance transnational TV series produced by Netflix or CBO. We live in a world of frequent mediated or real interaction with people from other cultures, countries, or backgrounds. This interaction makes Others, both real and imagined, more salient and more relevant in our understanding of who we are and who ‘others’ are. Such cultural consumption and indirect contact however do not necessarily imply openness or acceptance of other cultures or peoples. On the contrary, such multi-cultural tasting may trigger the urge to clarify one’s own identity and sense of belonging in a world of increased complexity and inter-cultural contact (Lemaine et al. 1978). In such a ‘liquid’ modernity (Bauman, 2000), people may feel free of their community ties but may also feel disoriented because they are left without stable reference points; nationalism and religion thus re-emerge with new force to fill the gaps.

In the case of nationalism, we have seen competing developments ranging from discourses of chauvinist, populist nationalism to ideologies of transnational, universalist solidarity and mixity (Triandafyllidou, 2020). We need to consider a spectrum ranging from plural forms of nationalism (that acknowledge differences, interact with them, and eventually embrace and synthesise new national configurations) to forms of neo-tribal reactive nationalism that is exclusionary, based on the construction of an authenticity and homogeneity that is organic and does not change. Neo-tribal and plural nationalism are of course ideal types, not black and white distinctions. These make more sense as the two extreme points of a continuum along which we can position the re-emerging nationalisms of today (Triandafyllidou, 2020).

The question, of course, that arises concerns the role of religion in this context of increased tensions between plural and neo-tribal forms of nationalism and how this relationship evolves in Central Eastern and Southeastern Europe – a region where emigration rather than immigration has been the main challenge in the past 30 years (Buchoswki 2020). It is my contention that religion can play out in either direction, as an element that makes the nation open up to other groups, on the basis of religious commonality or on the basis of respect for our joint humanity, universal solidarity and peace. At the same time religion can be used in exclusionary ways as a marker to differentiate and exclude minorities and migrants within the nation, labelling them as impossible to integrate because of their cultural and religious difference. It is worth looking a little closer into the relationship between religion and national identity as well as nationalism in the context of post-communist Europe, given the repression of religion and religious institutions under Communism and the intensive secularisation of society, as well as religion and religious institutions’ rebound into public and political life.

## Nation and religion in central eastern and southeastern europe

The political transition in Central Eastern and Southeastern Europe after 1989 has prompted significant scholarly research on the relationship between church and state, as well as between faith and nationalism. As Hoppenbrouwers (2002: 310) rightly points out though such a relationship could variably be one of competition and struggle, cooperation and fusion, or indeed of mutual exploitation, and finally of indifference. Hoppenbrouwers (ibid.) also points to the fact that state and church relations are seen differently within each of Christianity’s main traditions – notably Orthodoxy, Roman Catholicism and Protestantism – but such differences are more a matter of degree than absolute. In Europe, and particularly in Central Eastern and Southeastern Europe, churches have been organised as national entities as part of the nation formation processes, particularly at the time of Ottoman Empire’s dissolution. The gospel was seen as an element of the national culture that often helped distinguish from neighbours (e.g. between Croats and Serbs) who shared perhaps a common history, language, and culture (Triandafyllidou and Paraskevopoulou, 2002). Indeed, ethnic nationalism in the region has often been seen as one particularly marked by religious overtones. This is particularly true of the Orthodox churches which are actually the largest in this region.

More closely examining the historical relations between nationalism and the Orthodox religion, Malesevic (2019) and Kitromilides (2019) argue that Eastern Orthodox churches were initially inimical to nationalism, which they realised was a secular, liberal, and modernising project that undermined the shared cultural space that the Ottoman and earlier Byzantine empires offered for the everyday life of the Orthodox Christian population in the wider region. Nationalism was seen as a threat to the religious elites but also to the Christian Orthodox way of life (Kitromilides 2007). However, as the Ottoman Empire crumbled and independent nation-states emerged in Greece, Bulgaria, Serbia, and Romania, the Byzantine tradition of autocephaly was revived to serve nationalist purposes. The independence of Greece in 1832 was thus coupled with the creation of a separate Greek Orthodox Church. Similarly in 1872, the Bulgarian church proclaimed its autocephalus status (Malesevic 2019: 2).

Kitromilides (2019) aptly shows that the national organisation of Orthodox religious institutions in Central Eastern and Southeastern Europe and the nationalist view of religion as part and parcel of national identity was a project driven by state leaders for their own purposes rather than some sort of historical continuity with a pre-existing tradition, let alone a theological requirement. By contrast the close entanglement of nation and religion and of church and state in the region may be seen as quintessentially paradoxical from a religious tradition perspective (see Malesevic 2019 for a full discussion). Kalkandjieva (2011: 595-6) argues that the creation of autocephalous churches with clear territorial jurisdictions significantly transformed Christian Orthodoxy, imbuing it with the concept of phylethism despite its condemnation by the Great Local Synod in Constantinople in 1872 as a deviation from Orthodox ecclesiology. The linkages between church and state in those newly created states were further strengthened as national languages were adopted for the liturgy with a view of bringing believers closer to the church (Kalkandjeva: 596).

It is worth noting that in this regional context, relations between church and state never followed the principle of a ‘free church in a free state’ (Kalkandjieva, 2011: 602) as happened in western Europe with the concordats regulating the relationship between the Catholic Church and the modern nation-states.

While the establishment of Communist rule violently ruptured the development of nation formation concomitantly with national churches and nationalist religious ideologies, it did not introduce a true separation of church and state in Communist countries. Communist regimes were not secularist as in western Europe but rather militantly atheist. Confronted with strong national churches, they co-opted them with a view to using them to fuel patriotic sentiment (Knox 2007). The effort of uprooting religion was coupled with the control of the churches, which also helped undermine their role in opposition movements. This awkward relationship between the Communist regimes and the national churches – notably a relationship between repression and total control – needs to be taken into consideration when analysing the religion-nation and church-state relations in former Communist countries.

As Communism collapsed, significant socioeconomic and political transformation ensued, and some scholars have argued that this created not only a political but also a moral vacuum (Hoppenbrouwers, 2002: 307). Religious nationalism thus emerged in Central Eastern and Southeastern Europe as an answer to the search for meaning and for solidity (as opposed to the liquid modernity of Zygmunt Bauman (2000) or the irresistible lightness of being of Milan Kundera (2005)). Such religious nationalism was actually partly the product of political and institutional transformation. The new post-communist states offered public funding support to their churches to compensation for confiscated church property under Communism (Ferrari 2003). Constitutional laws in post-Communist countries also underscored the historical role of Orthodox churches in the preservation of the nation. Such emphasis and close links between church and state also aimed at taming the emergence and mobilisation of historical minority faiths as well as new religions that had been supressed under Communism (Pew 2017; Mate-Toth and Rosta 2017).

The rapid transition from an atheist state – albeit one that had co-opted the national churches – to a secular state led to a situation where the separation of church and state was incomplete. As the former Communist countries joined the EU in the 2000s, legislation adapted to European and international norms regarding human and religious rights and respect for minorities; in practice, however, states were quite concerned with religious minorities and the sprawling of new faiths. The transformation took a different route where the embrace between church and state and between nation and religion remained strong.

Finding the right balance between freedom of religion and respect for religious diversity has been complicated. These societies had not experienced a true separation of church and state at any stage of their history and, indeed, had been shaped by the strong relationship between majority religion and nation, and church and state, typical of Central Eastern and Southeastern European countries in the late 19th and early 20th century, as discussed earlier. The model of a dominant status of the Orthodox Church and Orthodoxy as a majority religion was in fact also preserved in Greece – which did not experience Communist rule – despite its formal adherence to the freedom of religion principle and related EU and international law (see also Magazzini et al., 2022). It is thus no surprise that post-Communist countries to this day struggle to find the right balance between the religious and the secular. The tensions become apparent when religious minorities and their rights are considered, as the prevalent role of Orthodox churches often undermines access and implementation of such rights (Magazzini et al., 2022). Orthodox churches in the region in fact see themselves as contributing to the development and consolidation of liberal democracy by reinforcing national unity and enriching citizens’ spiritual and moral life. Cooperation between Orthodox churches and Communist state security agencies in the past is, in this context, silenced or swept under the carpet (Kalkandjeva, 2011).

According to Hoppenbrouwers (2002), religious nationalism in Central Eastern and Southeastern Europe was more a symptom of the transition than an enduring phenomenon. While it has its roots in past historical relations, both at the identity and institutional levels – as also argued in this paper – it was more of a product of a difficult socio-economic and political transition that, on one hand, created important political and ontological uncertainties to the citizens of former Communist countries while also opening up a space for political leaders to co-opt churches for political purposes and for religious leaders to seek to re-establish their influence through their active involvement in politics.

Thus, writing 20 years ago, Hoppenbrouwers noted:

There seem to be four principal reasons why churches may espouse religious nationalism. First, believers and hierarchs alike may discern a missionary calling (..) in the nation. Second, (..) church leaders may use nationalistic themes in order to gain support not only among believers but from the powers that be as well. Third, the absence of any serious and systematic reflection on the past might lead them to substitute for rational discourse a religio-cultural ideology in which a spirituality of leadership, excellence or suffering is developing against a background of familiar or comforting imagery, music, arts and crafts (..) Fourth, a fear of western culture and its accomplishments may seduce people into thinking that an alternative to both the closed, repressive communist society and the open and pluralist society exists or should be developed [within the Christian tradition]. (..) I am inclined to see religious nationalism as an indicator of the intensity of the problems of transformation and therefore as a passing phenomenon. With the improvement of the economic and political situation extremist features will surely disappear (Hoppenbrouwers 2002: 315).

Perhaps what Hoppenbrouwers and other scholars two decades ago did not consider were the wider societal and geopolitical transformations happening within Europe that would shape further the relationship between religion and nationalism in Central and Eastern Europe. Thus while former Communist countries gradually joining the Council of Europe and some also the EU adopted the relevant legal norms and developed related laws – including being subjected to the scrutiny of the European Court of Human Rights (see also Anagnostou and Psychogiopoulou 2010) – new challenges emerged. As argued in the first section of this paper, these were not only persistent socioeconomic inequality both within and between EU countries but also new immigration and asylum seeker flows and a confrontation with both old cultural diversity (notably Roma populations) and with new cultural diversity (recent migrant and refugee arrivals). In the next section of the paper I shall discuss how responding to these new challenges has further transformed the relationship between nation and religion in Southeastern and Central Eastern Europe.

## Nationalising and politicising religion

Contributions in this Special Issue highlight the emergence of religious nationalism in a number of former Communist countries notably Bulgaria, Croatia, Hungary, Albania, Bosnia Herzegovina, and even Russia. While it goes beyond the scope of this paper to compare among these cases (see Sarkissian in this Special Issue for a systematic comparison), it is clear from the analyses provided that in all these countries religion has been developing a close relationship with illiberal nationalism at the expense of democracy and respect for diversity. While the discussion thus far on the relationship between nation and religion points to their closed interconnection and a rather ambivalent separation, particularly between Orthodox churches and the national state, why this relationship developed in further nationalist and illiberal overtones remains an open question. However, a closer look at the kind of religious nationalism emerging in Bulgaria (Iliyasov, n.d.) or Hungary (Vekony and Racius, n.d.) points to some important elements.

There is a rising right-wing populist authoritarian wave across the region that utilises religion and particularly Christianity (whether Orthodoxy or Catholicism) as a ‘nodal point’ (Laclau 2005), notably as a condensed set of meanings and signifiers that fix an exclusionary identity. Confronted with increased diversity and increased interconnectedness and interdependence, nationalism unfolds in this context in more neo-tribal than plural directions. Religion becomes an important element in this mix, given its strong role and presence in these countries and its continuous entanglement with politics. It thus lends itself to becoming an important identity marker in the absence of the Left and Right ideological struggle in the post-1989 context. As the socio-economic and political transition continued to be incomplete and painful for society and a large segment of the population, leading to disenchantment with the free market economy and parliamentary democracy, religion offered the possibility of a new anchoring – refuting (at least partly) Western liberal and plural democratic values. Religion as an ascriptive identity marker, a historical factor, and a political element provides the perfect ingredient for nativism and populism and their manichaeistic, dichotomous, and oppositional, view of the world as divided between ‘us’ and ‘them’, locals and foreigners, people and elites.

Nativism emerged as a political movement in the early 19th century in the United States in response to mass immigration from Europe (Betz 2017) – although some argue that it appeared as early as the 1600s once the first colonists established themselves in the new continent (Anbinder, 2006: 177-178). Literature on nativism is scant for a good part of the 20th century until the concept resurfaced to express anti-immigrant sentiment in the last two decades (Anbinder, 2006). According to Anbinder, nativism has taken different connotations at times, focusing on specific ‘racial’, ethnic, or other groups such as Asians or Jews, and is mostly related to status anxiety rather than economic grievances. It has however always had a clear concern with restricting immigration (Anbinder, 2006; Betz 2017).

More recent scholarly work on nativism has emphasised the etymology of the term: it is about the preference of the ‘native’ exclusively on the grounds of ‘being native’ and about the rejection of anything new and foreign and hence of immigrants. Nativism can be seen as closely related to populism in its attention to the lay people and in its nostalgic reference to an idealised past (Taggart 2000). Recent literature has distinguished between different types of nativism depending on where the exclusionary focus of nativist discourses and movements has been: racialised nativism (Lippard 2011), cultural nativism (Davidson and Burson 2017), symbolic nativism (Schildkraut 2003), or economic nativism (Anbinder 1992). Nativism need not be conservative; it can also be progressive (Taylor 2000). Nonetheless, in all its versions nativism is predicated on bringing people together in a sort of nativist exclusionary solidarity and identity against immigrants.

Populism, like nativism, is built on the basis of an opposition between the people, seen as a homogenous organic community, and the elites, identified as external to the community, corrupt, and oppressive. As Shils noted, in populism the will of the people enjoys absolute priority over any other principle, right, or institutional standard; the people are seen as the source of both justice and morality (Shils 1956: 97). Thus, the notions of justice or morality are almost decoupled from their content and made independent from the rule of law. Suffice that the will of the people is expressed: that is all that matters. Populism is actually often anti-democratic even if it can thrive in democratic systems as populist parties may be electorally successful and gain power. Urbinati (2014: 109) notes that populism seeks to overcome pluralism and use partisanship to create a unified opinion that is supposed to represent the ‘whole people’.

One might ask though why Central Eastern and Southeastern European countries are more open to nativist and populist currents and the role of religion and religious nationalism in those. There are three reasons. First, the transition to democracy, rule of law. and a free market economy has for all the countries been particularly painful, prolonged, and at times partial thus undermining support for democracy (Adam and Bozoki 2016; Rupnik 2018). Second the institutional transformation was hamstrung by an incomplete implementation of the rule of law at the level of everyday politics or the economy (Kalkandjeva 2011). Last but not least, religion remained important both at the identity level as a marker in the absence of valid competing ideological positions and because of its re-emergence post-1989 as a spiritual and cultural reference point as well as a political and institutional element (as argued in this paper, see also Lamour 2022).

Lamour (2022: 320-1) in fact asks whether we are witnessing the politicisation of religion or the sacralisation of politics. He identifies two main features of the former, notably when political discourse and actions are informed by the faith, holy books, or God – in other words when populist leaders assert that their vision aims at achieving God’s will or protecting the faith. A second strand consists of emphasising religion as an element of identity, and particularly one that is under threat, for instance from an imminent ‘Muslim invasion’ or in line with ‘replacement theories’. The sacralisation of politics, according to Lamour, refers to the affective investment of political discourse through references to myths, rites, and symbols. This is a feature typical of totalitarian regimes such as Fascism or Nazism or actually Communism (Lamour, 2022: 321)

Radical right populist, authoritarian, and identitarian political parties and movements do not only exist in Central Eastern Europe – they are particularly strong also in western and Nnorthern Europe as the recent ascent to power of the neo-fascist Brothers of Italy in Italy has shown, as well as the significant electoral successes of Marine Le Pen and the Swedish radical right. These parties utilise religion too in their nativist and populist discourses with a view to exclude migrants and minorities (see also Norris and Inglehart 2019; Schworer and Romero-Vidal, 2020). As Zuquete (2017) argues, religion lends itself well to support the moral claims of populism by supporting an antagonistic moral discourse between the good and moral people and the corrupt and immoral elite. Interestingly, it is these populist elites who appear as messianic saviours to protect the nation and steer it to safety.

The cases of Poland and Hungary are indicative of these dynamics. In either country the population’s actual experience with Muslim migrants or refugees is mostly empirically absent and rather imagined. However, the refugee emergency of 2015-2016, manipulated by a populist and nationalist political leadership (Krzyzanowksi 2018), has led to a ‘phantom Islamophobia’, as Buchoswki (2020: 74) calls it, that fits nicely with representations of the Oriental Other. The ethnoreligious composition of different Central Eastern and Southeastern European countries varies as some are ethnically and religiously homogenous like Poland, while others include significant national minorities (like Hungary or Slovakia or the Baltic states) yet others like Bulgaria have significant Muslim minorities. And yet what is common is the securitisation of religion on the basis of an imagined Muslim threat that resonates with the narrative of state- and nation-formation (e.g. in Bulgaria or Greece) or with generalised orientalising xenophobic stereotypes (as in Poland or Slovakia). Thus a nationalist religious populism promptly emerges in all these cases. While these identity and political dynamics develop in parallel in western and Central Eastern or Southeastern European countries, their success may be stronger and their power higher in the latter because of the level of socio-economic challenges that these have been confronted with over the last 30 years and because of their historical legacies and experiences of state-church entrenchment that Communist rule manipulated rather than dissolved. While we may also consider the transnational linkages among populist and nativist movements across Europe, recent research (Suchánek and Hasman, 2022) shows how religion is an important factor alongside more context-specific elements.

## Concluding remarks

This paper adopts an interactive perspective in considering how nationalism as well as the relationship between nationalism and religion evolve. While past theories have paid more attention to the content of national identity and the role of religion within it, I am rather focusing on how this relationship developed and transformed in a broader socioeconomic and political context of change within both a given country or region but also globally. Earlier studies on the relationship between nationalism and religion in Central Eastern and Southeastern Europe have pointed to the importance of historical identity factors, institutional entanglements, an incomplete separation of both church and state, and strong historical and cultural linkages between national identity and faith. More recent studies on the rise of populist and authoritarian religious nationalism have additionally looked at the political, institutional, and identity dynamics characterising former Communist countries. Paying attention to their being young democracies – whose citizens have been increasingly disillusioned by democracy and the transition to a liberal society and a free market economy – many studies have also focused on charismatic populist leaders and have seen religion as purely instrumental.

This paper adds to this line of thought. It argues that these relations do not only develop in a specific national context with particular local and national dynamics but in response to broader transformations stemming from European integration and social changes characterising liquid modernity (Bauman 2000). While the re-emergence of old ‘solidities’ (ibid.) may be a general phenomenon, in Central Eastern and Southeastern Europe this process assumes overtones of religious nationalism because of past and contemporary dynamics.
